# CD1d- and MR1-Restricted T Cells in Sepsis

**DOI:** 10.3389/fimmu.2015.00401

**Published:** 2015-08-12

**Authors:** Peter A. Szabo, Ram V. Anantha, Christopher R. Shaler, John K. McCormick, S.M. Mansour Haeryfar

**Affiliations:** ^1^Department of Microbiology and Immunology, Western University, London, ON, Canada; ^2^Division of General Surgery, Department of Medicine, Western University, London, ON, Canada; ^3^Centre for Human Immunology, Western University, London, ON, Canada; ^4^Lawson Health Research Institute, London, ON, Canada; ^5^Division of Clinical Immunology and Allergy, Department of Medicine, Western University, London, ON, Canada

**Keywords:** CD1d, MR1, NKT cell, MAIT cell, LPS, α-galactosylceramide, infection, sepsis

## Abstract

Dysregulated immune responses to infection, such as those encountered in sepsis, can be catastrophic. Sepsis is typically triggered by an overwhelming systemic response to an infectious agent(s) and is associated with high morbidity and mortality even under optimal critical care. Recent studies have implicated unconventional, innate-like T lymphocytes, including CD1d- and MR1-restricted T cells as effectors and/or regulators of inflammatory responses during sepsis. These cell types are typified by invariant natural killer T (*i*NKT) cells, variant NKT (*v*NKT) cells, and mucosa-associated invariant T (MAIT) cells. *i*NKT and *v*NKT cells are CD1d-restricted, lipid-reactive cells with remarkable immunoregulatory properties. MAIT cells participate in antimicrobial defense, and are restricted by major histocompatibility complex-related protein 1 (MR1), which displays microbe-derived vitamin B metabolites. Importantly, NKT and MAIT cells are rapid and potent producers of immunomodulatory cytokines. Therefore, they may be considered attractive targets during the early hyperinflammatory phase of sepsis when immediate interventions are urgently needed, and also in later phases when adjuvant immunotherapies could potentially reverse the dangerous state of immunosuppression. We will highlight recent findings that point to the significance or the therapeutic potentials of NKT and MAIT cells in sepsis and will also discuss what lies ahead in research in this area.

## Preamble

Sepsis is a life-threatening syndrome typically associated with early hyperinflammation, immunosuppression in its protracted phase, and a continuum of organ dysfunction abnormalities. It is a significant cause of death across all age groups and in both developed and developing countries. It also negatively affects the quality of life among survivors. Sepsis is usually a consequence of infection although sterile tissue damage inflicted by non-infectious causes or conditions, such as pancreatitis, ischemia–reperfusion injury, and cancer may also lead to sepsis (1). In this article, we will only focus on the syndrome caused by disproportionate, excessive, or sometimes defective host responses to infection. We will provide a general overview of sepsis, its epidemiology, prognosis, management, and immunopathogenesis. We will briefly discuss experimental immunotherapeutic strategies tested in animal models of sepsis or used in clinical trials. Many such strategies have targeted antigen-presenting cells (APCs) and conventional T cells or their products, such as inflammatory cytokines, albeit with little success. Recent progress in our understanding of natural killer T (NKT) cell and mucosa-associated invariant T (MAIT) cell responses to infection and their regulatory functions may open a new front in our fight against sepsis. These unconventional T cells respond rapidly to infection by secreting large quantities of pro- and/or anti-inflammatory cytokines, thereby controlling the effector functions of numerous other cell types belonging to both innate and adaptive arms of immunity. Also importantly, NKT cells can be easily manipulated by “disease-tailored” synthetic glycolipids. Therefore, the quick and wide-ranging actions of NKT cells, and potentially of MAIT cells, may be exploited to the host’s benefit in different forms or stages of sepsis. We will review NKT and MAIT cell functions in antimicrobial immunity and highlight recent findings on these cell types in the context of sepsis.

## NKT Cells: A Brief Overview

Natural killer T cells are innate-like T lymphocytes with impressive immunomodulatory properties. They express glycolipid-reactive αβ T cell receptors (TCRs) along with several characteristic markers of NK cells (e.g., mouse NK1.1 and human CD161) (2, 3). NKT cells develop in the thymus where they are positively selected by CD1d^+^CD4^+^CD8^+^ thymocytes and consequently become “CD1d-restricted” (4). As such, CD1d-deficient mice are devoid of NKT cells (5). CD1d is a monomorphic major histocompatibility complex (MHC) class I-like glycoprotein that is highly conserved across mammalian species (6). It is a member of the CD1 family of lipid antigen (Ag)-presenting molecules (7, 8). The CD1 family in human has five members, namely CD1a–e, while rodents only express CD1d. Murine and human CD1d can present normal self- and tumor-derived lipids as well as microbial glycolipids to NKT cells. The discovery of CD1d restriction led to the invention of glycolipid-loaded CD1d tetramer reagents enabling accurate tracking, enumeration, and phenotypic and functional analysis of NKT cells (9–11).

The major subset of NKT cells is defined by the expression of a canonical or invariant TCR (*i*TCR) with a unique α chain rearrangement (Vα14–Jα18 and Vα24–Jα18 in mice and humans, respectively), which is paired with one of only a limited choices of β chains (Vβ8.2, Vβ2 or Vβ7 in mice and Vβ11 in humans). These cells are called type I or invariant NKT (*i*NKT) cells (2, 3). Two phenotypically distinct subpopulations of *i*NKT cells have been identified in mice, the CD4^+^CD8^−^ subset and the double-negative (CD4^−^CD8^−^) subset (12). An additional CD8α^+^ subset exists in humans (13). *i*NKT cells constitutively express CD69, CD25, and CD44 on their surface, which is consistent with their “partially-activated” or “memory-like” status even in germ-free mice (14) and in human cord blood (15).

*i*NKT cells are present at low frequencies in the circulation and in various tissues including bone marrow, thymus, spleen, and lymph nodes. However, they are abundant in the mouse liver and in the human omentum (16). The prevalence of *i*NKT cells varies considerably among different individuals for reasons that are currently unknown. Also importantly, *i*NKT cell subsets found in different anatomical locations exhibit functional or even transcriptional heterogeneity. For instance, interleukin (IL)-4- and IL-13-producing human peripheral blood *i*NKT cells fall exclusively within the CD4^+^ subset, whereas *i*NKT cells that synthesize tumor necrosis factor (TNF)-α and interferon (IFN)-γ upon *ex vivo* stimulation can be either CD4^+^ or double negative (17). Another example is the case of adipose tissue *i*NKT cells that secrete IL-10, impart an anti-inflammatory phenotype to macrophages, and control the expansion and suppressor function of regulatory T (Treg) cells (18). Moreover, adipose tissue *i*NKT cells lack promyelocytic leukemia zinc finger (PLZF), a transcription factor otherwise regarded as a “master regulator” of *i*NKT cell effector functions (19).

*i*NKT cells are armed with cytotoxic effector molecules such as perforin, granzymes, TNF-α, Fas ligand, and TNF-related apoptosis-inducing ligand (TRAIL), and may be able to lyse neoplastic or infected cells directly (20–22). However, they are best known for their immunomodulatory functions mediated by the early production of pro- and/or anti-inflammatory cytokines. *i*NKT cells can thus transactivate numerous downstream effector cell types including natural killer (NK) cells, macrophages, dendritic cells (DCs), conventional CD4^+^ and CD8^+^ T cells, and B cells. They are rapid producers of enormous quantities of T helper (Th)1-, Th2-, and Th17-type cytokines, although Th9- and Th10-like *i*NKT cells have also been described (23, 24). The constitutive presence of pre-formed messenger RNA (mRNA) encoding at least some of such cytokines in *i*NKT cells explains the rapidity with which they are released (25).

The identity of endogenous CD1d ligand(s) that participate in positive selection and also perhaps in peripheral maintenance of *i*NKT cells remains ill-defined and controversial. *i*NKT cells can recognize and respond to certain glycolipids present in various microbes, including but not limited to *Novosphingobium* spp., *Ehrlichia* spp., *Borrelia burgdorferi*, *Streptococcus pneumoniae*, and *Streptococcus agalactiae* (26–28). Of note, the latter pathogen, which is often referred to as group B streptococcus, is a common cause of neonatal sepsis.

Of all exogenous glycolipid agonists of *i*NKT cells, α-galactosylceramide (α-GalCer) has been used most extensively, not only as a research tool but also in clinical trials for cancer and viral diseases (29). α-GalCer was initially isolated from an extract of a marine sponge called *Agelas mauritanius* (30), and is believed to have originated from microbes co-existing in a symbiotic relationship with this sponge. Until recently, α-GalCer was considered to be a merely exogenous and unnatural glycolipid given the presence of only one glucosylceramide synthase and one galactosylceramide synthase in mammalian species, both of which are β-transferases. However, a recent report has demonstrated the presence of endogenous α-anomeric glycolipids including α-GalCer in mammals, due perhaps to the operation of an “unfaithful” enzyme or a novel, as-yet-unidentified pathway (31).

α-GalCer and its analogs possess a lipid tail that can be buried deep inside the hydrophobic pocket of CD1d, while their galactose head protrudes out of CD1d to be contacted by the *i*TCRα chain (32). The length and composition of acyl and phytosphingosine chains of synthetic α-GalCer analogs impact the binding affinity of α-GalCer:CD1d:*i*TCR interactions (33), which partially determines the type of cytokines that an activated *i*NKT cell will secrete. For example, OCH is a sphingosine-truncated derivative of α-GalCer with Th2-skewing characteristics (34). We have successfully used this glycolipid to delay Th1-mediated cardiac allograft rejection (35), to prevent or cure citrulline-induced autoimmune arthritis (36), and to reduce the severity of intra-abdominal, polymicrobial sepsis (37) in mouse models. Another Th2-favoring agonist of *i*NKT cells is C20:2, an α-GalCer analog with a short fatty acyl chain containing two unsaturation sites at carbon-11 and -14 positions (38). C20:2 is reportedly superior to OCH in polarizing human *i*NKT cells. Th1-biasing ligands of *i*NKT cells can be exemplified by a C-glycoside analog of α-GalCer, also known as α-C-GalCer, which potentiates IL-12 and IFN-γ production in mice (39). Therefore, α-C-GalCer may be useful in adjuvant glycolipid immunotherapy of cancer and infectious diseases.

Cell membrane location of glycolipid loading onto CD1d and its presentation within or outside lipid rafts (40), the type of CD1d^+^ APCs involved (41), the presence and intensity of costimulatory and danger signals transmitted or exchanged (29), and the cytokine milieu in which *i*NKT cell priming occurs are among other important factors that shape the cytokine profiles of *i*NKT cells. Remarkably, mouse *i*NKT cells can recognize human CD1d and *vice versa* (6), and *i*NKT cells from either species are responsive to α-GalCer. Therefore, at least some of the findings obtained in mouse models of CD1d-mediated *i*NKT cell activation are likely to be translatable to the clinic.

*i*NKT cells can also be activated in the absence of exogenous glycolipids. During infection, microbial components may engage pattern recognition receptors (PRRs), such as Toll-like receptors (TLRs) on APCs, thus resulting in secretion of IL-12 and IL-18. Together, these cytokines trigger indirect activation of *i*NKT cells (42, 43), which is often dependent upon the presence of CD1d. This indicates an intriguing but poorly understood role for endogenous lipids in the context of antimicrobial immunity. A combination of IL-12 and IL-18 can also reportedly induce *i*NKT cell responses in a truly *i*TCR-independent fashion (44). We recently reported that group II bacterial superantigens (SAgs) can directly activate *i*NKT cells in a CD1d-independent manner (45). Therefore, *i*NKT cells may serve as effectors and/or regulators of early cytokine responses to bacterial SAgs.

Type II or variant NKT (*v*NKT) cells are CD1d-restricted cells with a relatively diverse αβ TCR repertoire (3, 11). They exhibit reactivity with certain self lipids, but not with α-GalCer (46). Compared with *i*NKT cells, *v*NKT cells are less frequent in mice but more prevalent in humans (47). A major fraction of *v*NKT cells can recognize sulfatide, a self glycolipid that is highly enriched in the central nervous system, kidney and liver (48). Several other endogenous lipids, including but not limited to β-d-glucopyranosylceramide (β-GlcCer), have also been recently discovered to activate *v*NKT cells (49). Given the relative promiscuity of *v*NKT cell Ag receptors, it is not too far-fetched to envisage scenarios where *v*NKT cells recognize microbial lipids cross-reactive to self components. In addition, infection may lead to the release of self lipids in sufficient quantities to induce *v*NKT cell activation.

Our overall understanding of *v*NKT cell responses in health and disease is limited. This is in large part due to a lack of firm molecular markers, stable reagents and direct methods to detect and characterize *v*NKT cells. Sulfatide-loaded CD1d-tetramer reagents have been generated (46). However, they are not popular due to their low stability and high background staining. In addition, there is no mouse model of pure *v*NKT cell deficiency. CD1d^−/−^ mice are devoid of both *i*NKT and *v*NKT cells since CD1d is required for the positive selection of both cell types in the thymus (50). Experimental evidence indicates that *v*NKT cells have an activated phenotype and depend on PLZF for their development (49), and that *v*NKT and *i*NKT cells may exert opposing functions with broad implications for antitumor responses (51) and antimicrobial immunity (52, 53).

## MAIT Cells and Their Roles in Microbial Immunity

MAIT cells are another evolutionarily conserved subset of innate T lymphocytes that have captured the attention of the immunological community in the past few years (54, 55). MAIT cells develop in the thymus where they rearrange their semi-invariant TCR with a characteristic Vα19–Jα33 and Vα7.2–Jα33 TCRα chain in mice and humans, respectively (56, 57).

Similar to NKT cells, MAIT cells are positively selected by CD4^+^CD8^+^ thymocytes (58). However, their selection requires the expression of MHC-related protein 1 (MR1), as opposed to CD1d, on thymocytes. Accordingly, MR1-deficient mice lack MAIT cells in their T cell repertoire (59). MR1 is a monomorphic, non-classical MHC I molecule that is markedly conserved among various mammals (60–62). There is 90% sequence homology between mouse and human MR1 ligand-binding domains and a high degree of functional cross-reactivity, which is highly reminiscent of cross-species CD1d conservation.

MAIT cells are infrequent and immature in the human fetal thymus (63). Their maturation is accompanied by a gradual, post-thymic acquisition of PLZF expression and the ability to secrete IFN-γ and IL-22 upon exposure to microbes in mucosal layers. A PLZF-expressing CD161^high^CD8^+^ population is detectable in human cord blood, from which Vα7.2^+^ MAIT cells emerge in adults (64).

MAIT cells are severely depleted in B cell-deficient patients and mice, and are also entirely absent in the peripheral tissues of germ-free mice (59), indicating that B cells and commensal microflora are essential for MAIT cell peripheral maintenance/expansion. Therefore, it is not surprising that MAIT cells preferentially accumulate in the mucosal compartments, such as the gut lamina propria, hence their denomination. MAIT cells are also present in other tissues. In human, they are particularly abundant in peripheral blood and can comprise up to ~50% of all T cells in the liver (65, 66). There are far fewer MAIT cells in mice than in humans. This, together with other differences between the two species (67), indicates that caution needs to be exercised in extrapolating experimental data from mice to human conditions.

Until recently, there was no single reagent to directly detect mouse MAIT cells. In addition, human MAIT cells have been commonly defined as CD3^+^Vα7.2^+^CD161^+^. However, recent identification of a MAIT cell Ag, namely reduced 6-hydroxymethyl-8-d-ribityllumazine (rRL-6-CH_2_OH), led to the development of MR1 tetramer reagents loaded with this compound to accurately identify mouse and human MAIT cells (68). Once widely available, these reagents will undoubtedly boost research in the area of MAIT cell biology. Human peripheral blood MAIT cells are CD45RA^−^CD45RO^+^CD62L^low^CD95^high^, which is consistent with an effector memory phenotype (65). They also express the receptors for IL-12, -18, and -23. Human hepatic MAIT cells have a more activated phenotype and express elevated levels of CD69 in comparison with their blood counterparts (66). They are also human leukocyte Ag (HLA)-DR^+^ and CD38^+^. This may be due to continuous exposure to microbial Ags accessing the liver from the gut through the portal system.

MAIT cells bridge innate and adaptive arms of immunity to microbial intruders. They quickly amass in sites of infection where they can keep pathogens in check. For instance, in a mouse model of pulmonary infection with *Francisella tularensis*, MAIT cells reduce bacterial burden in the lungs and prevent mortality from infection even in the absence of conventional T cells (69). They can produce inflammatory cytokines such as IFN-γ, IL-17, and TNF-α readily, amply and promptly after TCR stimulation (54, 55). Human MAIT cells express granzymes A and K, and are able to kill infected cells (70). They were shown to lyse, in an MR1-dependent fashion, epithelial cells infected by the intestinal pathogen *Shigella flexneri* (71), and THP1 monocytic cells infected by *Escherichia coli* (*E. coli*) (70). MAIT cells are responsive to a variety of bacteria and yeasts including *Lactobacillus acidophilus*, *Klebsiella pneumoniae*, *Pseudomonas aeruginosa*, *Staphylococcus aureus (S. aureus)*, *Staphylococcus epidermidis*, *Candida albicans*, *Candida galbrata*, and *Saccharomyces cerevisiae*. A limited number of studies have utilized MR1-deficient mice to explore the antimicrobial potentials of MAIT cells *in vivo*. The ability to control infection with *Klebsiella pneumoniae*, *Mycobacterium bovis* bacillus Calmette–Guérin (BCG), or *Francisella tularensis* was found to be impaired in MR1-deficient mice (69, 72, 73).

McCluskey’s and Rossjohn’s research teams discovered that vitamin B metabolites represent a class of MR1-restricted Ags (74). A folic acid (vitamin B9) metabolite called 6-formyl pterin (6-FP) was found to bind MR1 without stimulating MAIT cells. In contrast, MR1 ligands derived from the riboflavin (vitamin B2) biosynthesis pathway could activate MAIT cells. Of note, this pathway is operational in all of the microorganisms that activate MAIT cells, but not in those that reportedly fail to do so.

To confirm that the riboflavin pathway supplies human MAIT cell ligands, Corbett et al. mutated various enzymes of the riboflavin operon in the Gram-positive bacterium *Lactococcus lactis* followed by testing the MAIT cell-activating capacity of the mutants (75). This approach led to the identification of 5-amino-6-d-ribitylaminouracil (5-A-RU), an early intermediate of the riboflavin pathway, as a key compound in generating MAIT cell “neo-antigens.” Through non-enzymatic interactions, 5-A-RU forms simple adducts with small molecules arising from other metabolic pathways (e.g., glycolysis), such as glyoxal and methylglyoxal, thus giving rise to 5-(2-oxoethylideneamino)-6-d-ribytilaminouracil (5-OE-RU) and 5-(2-oxopropylideneamino)-6-d-ribytilaminouracil (5-OP-RU), respectively. MR1 in turn captures, stabilizes, and presents these neo-antigens to MAIT cells. Recent work from Olivier Lantz’s laboratory demonstrated that most, if not all, mouse MAIT cell ligands harbored by the Gram-negative bacterium *E. coli* are also related to the riboflavin pathway (76). MR1-mediated activation of mouse MAIT cells was most robust upon stimulation with a mixture of 5-A-RU and methylglyoxal, and also detectable when a combination of 5-A-RU and glyoxal was used. This study also reported the synthesis of a new 6-FP variant in which the amine and the formyl group are blocked. This compound could efficiently inhibit the activation of MAIT cells by semipurified soluble bacteria (SPB) or by 5-A-RU plus methylglyoxal, and may therefore represent a new class of inhibitors of MAIT cell activation. Finally and importantly, *in vivo* activation of MAIT cells was demonstrated for the first time when *i*Vα19 transgenic mice on a Cα^−/−^ background, which harbor many MAIT cells, were directly injected with the SPB fraction from riboflavin-sufficient *E. coli* or with a mixture of 5-A-RU and methylglyoxal. Interestingly, administration of 5-A-RU alone failed to activate MAIT cells, which may be probably due to its instability and/or low bioavailability for interaction with small metabolites and loading onto MR1 (76).

Mammals do not synthesize riboflavin, but host-derived metabolites could potentially generate adducts with 5-A-RU of bacterial origin (75). MR1-restricted recognition of the formed neo-antigens may be considered a new mechanism of self–non-self discrimination, especially in mucosa-associated lymphoid tissues. MR1 ligands are ubiquitous and present in many bacteria, including commensals. In addition, they can readily diffuse across epithelial barriers (55). Therefore, how MAIT cell activation is controlled *in vivo* remains enigmatic at this point.

MR1-independent responses can also be mounted by MAIT cells. The *in vitro* response of MAIT cells to BCG-infected cells is an example (73). Moreover, MAIT cells can produce IFN-γ when cultured with a combination of IL-12 and IL-18 in the absence of TCR triggering (77). Therefore, bystander activation of MAIT cells may occur during infection with viral pathogens or other germs that do not harbor MR1 ligands.

## Sepsis

### Definitions and epidemiology

Although sepsis is often discussed in the context of intensive care in modern settings, the syndrome is almost as old as medicine itself. Derived from the Greek *sipsi* meaning “make rotten,” the term sepsis was first coined by Hippocrates (460–370 BC) to describe the unpleasant process of organic matter putrefaction (78). Avicenna (980–1037 AD), the great Persian physician/scientist/philosopher, noted the frequent coincidence of blood putrefaction, what is known today as septicemia, and fever in the aftermath of surgery (79). The centuries that followed witnessed important discoveries linking germs to a wide array of disorders including sepsis. However, the germ theory of disease failed to fully explain the pathogenesis of sepsis since many patients succumbed to it despite successful eradication of the microbial intruder(s). Therefore, the host response to the germ, and not the germ *per se*, was proposed to drive the pathogenesis of sepsis (80).

The modern terminology for sepsis and its sequelae was standardized during an American College of Chest Physicians/Society of Critical Care Medicine Consensus Conference in 1991 (81). Accordingly, sepsis is defined as documented or suspected infection accompanied by at least two of the following abnormalities: (i) a body temperature of >38°C or <36°C; (ii) a heart rate of >90 beats/min; (iii) a respiratory rate of >20 breaths/min or PaCO_2_ of <32 mm Hg; (iv) a blood leukocyte count of >12,000/mm^3^ or <4,000/mm^3^, or detection of >10% immature neutrophils (*aka*. band cells) in the leukocyte differential count. The panel of experts recommended the application of the term “severe sepsis” when sepsis is further complicated by organ dysfunction, perfusion abnormalities (e.g., lactic acidosis, oliguria, acute alteration in mental status), or hypotension (a systolic blood pressure of <90 mm Hg or a reduction of ≥40 mm Hg from the baseline in the absence of other causes of hypotension). It needs to be noted that the terms “sepsis” and “severe sepsis” have often been used interchangeably. Finally, a severely septic patient should be classified as having “septic shock” when her/his hypotensive state is refractory to fluid resuscitation.

Sepsis is a leading cause of death following hospitalization and represents a major challenge in the management of critically ill patients in non-coronary intensive care units (ICUs) (82). It is estimated that 25% of patients who develop severe sepsis die during hospitalization, and septic shock is associated with mortality rates approaching 50% (83). Alarmingly, the incidence of severe sepsis is on the rise (84). Of equal importance, sepsis worsens the quality of life among survivors and increases their risk of morbidity and early death. In fact, the 5-year mortality rate in the sepsis survivor pool can be as high as 75% (84).

### Risk factors and prognosis

In general, the prognosis of sepsis is dependent upon demographic, socioeconomic, and iatrogenic factors in addition to the patient’s medical history, immunological, nutritional and overall health status, and the type of microorganism(s) involved in triggering or perpetuation of sepsis (79, 85). For instance, being over 65 years of age, being a male, being a nursing home resident, being in a poor nutritional state, having low household income, or receiving treatment in a non-teaching hospital predisposes to sepsis and to its elevated severity. Several studies have found that age is an independent predictor of mortality from sepsis (86–88). However, the elderly are vulnerable to sepsis also due to a higher likelihood of pre- or co-existing morbidities (e.g., diabetes and cardiovascular problems) requiring medication, malnutrition, repeated and/or prolonged hospitalizations, decline in immunity, and functional restrictions (89). Some of the above factors are taken into account in calculation of Mortality in Emergency Department Sepsis (MEDS) score to predict 1-year mortality (85).

Adverse iatrogenic factors include steroid therapy and immunosuppression prior to surgery and a need for multiple operations (79). Invasive devices such as urinary and intravenous catheters and breathing tubes also increase the risk of sepsis. The main predisposing factor for urinary tract infections, which are the most frequent nosocomial infections in surgical patients, is the usage of an indwelling urinary catheter. Vascular catheters, especially central venous catheters, are also common vehicles for nosocomial infections caused by Gram-positive skin commensals.

It cannot be overstated that the prognosis of sepsis is also determined by the speed with which the diagnosis is established and proper management strategies implemented. The earlier the treatment is started, the more favorable the outcome will be.

### Clinical management

Despite advances in our understanding of sepsis at organismal, cellular and molecular levels, not even a single drug is approved as a mechanism-based treatment option for sepsis. The clinical guidelines established by the Surviving Sepsis Campaign (SSC), an international consortium of professional societies committed to reducing mortality from severe sepsis and septic shock, are organized into two “bundles,” each comprising a select but non-specific set of care elements distilled from evidence-based practice (90). The initial “resuscitation bundle” should be applied within 6 h after the patient’s presentation to prevent or resolve cardiorespiratory insufficiency and to combat the immediate threats posed by uncontrolled infection(s). Hemodynamic resuscitation is achieved by administration of intravenous fluids and vasopressors while oxygen therapy and mechanical ventilation can also be supplied as needed. The timely management of infection requires obtaining blood cultures before broad-spectrum antibiotic therapy is launched as well as source control (e.g., drainage of pus). The subsequent “management bundle” is typically accomplished in the ICU where the attention is shifted toward monitoring and supporting vital organ functions and avoiding complications. In addition, the efficacy of antibiotic therapy is evaluated for potential de-escalation to prevent the emergence of microbial resistance and to lower the risk of drug toxicity (90, 91).

A recent meta-analysis of 13 randomized controlled trials has demonstrated that early goal-directed therapy, which is perhaps best exemplified by the SSC-recommended resuscitation bundle, reduces overall mortality from sepsis when initiated within the first 6 h (92). This should reinforce the notion that there usually exists a short window of opportunity in which current management strategies or novel future therapies are expected to be most effective.

### Immunopathogenesis and immunosuppression in sepsis

Disproportionate or dysregulated immune responses to infection constitute a major culprit in sepsis-related death. Sepsis is no longer considered a merely or even mainly hyperinflammatory syndrome. Rather, in “sepsis-prone” individuals and conditions, infection triggers a highly complex response that is variable in proportion or in pro- versus anti-inflammatory nature depending upon the pathogen load and virulence, genetic and other host factors including age and co-morbidities, and the time point at which the response is evaluated (91). Pro-inflammatory responses mounted in septic patients help eradicate the inciting microbe(s) but may cause collateral organ damage. On the other hand, anti-inflammatory and immunosuppressive mechanisms contribute to tissue recovery but also make the patient susceptible to secondary infections and opportunistic pathogens, especially during protracted sepsis (93).

The pioneering studies of Tracey et al. in the mid-1980s revealed that many deleterious features of endotoxin administration to rats could be simulated by human cachectin (*aka*. TNF) (94) and that cachectin-neutralizing antibody F(ab′)_2_ fragments could prevent acute and otherwise lethal septic shock in *E. coli*-infected baboons (95). We now know that acute septic shock, which occurs in a relatively small fraction of patients with sepsis, is indeed a dangerous immunopathology mediated by an overly exuberant TNF response (91). TNF-α and other pro-inflammatory cytokines including IL-1β and IL-6 and chemokines like IL-8 are released from activated macrophages and other APCs after they sense the presence of invading microbes by PRRs (e.g., TLRs) and phagocytose them. This in turn leads to neutrophil mobilization, lymphocyte activation, and more pro-inflammatory cytokine (e.g., IFN-γ) secretion. These cytokines limit microbial infections, but their elevated levels are associated with a poor outcome in sepsis (96, 97). The pleotropic cytokine IL-3 was recently found to be an upstream orchestrator of inflammation in the early phase of polymicrobial sepsis modeled by the cecal ligation and puncture (CLP) procedure in mice (98). In addition, retrospective and prospective analyses of plasma IL-3 in septic patients linked heightened levels of this cytokine to a poor outcome.

Both pro- and anti-inflammatory processes get underway promptly after the initiation of sepsis. A hyperinflammatory “cytokine storm” dominates the initial phase in many patients and accounts for death within the first 3 days from septic shock and multiple organ failure in a substantial fraction of patients (99). However, more than 70% of deaths due to sepsis occur after the first 3 days, with many occurring weeks later. One needs to keep in mind that many, if not most, epidemiological studies on sepsis have been conducted in developed countries with an aging population and advanced ICU facilities. Therefore, the reported decline in mortality rates of early sepsis is likely owed to better management protocols and also perhaps a reflection of immunosenescence in the elderly.

Death during protracted sepsis is sometimes the result of the family’s decision to withdraw aggressive support measures to switch to palliative care for patients with severe co-morbidities and a slim chance of recovery. However, the fact remains that with or without such decisions, many patients in this phase succumb to stubborn infections that are difficult to resolve even with broad-spectrum antimicrobial therapy and infection source control (100). In a retrospective review of macroscopic autopsy findings, approximately 77% of surgical ICU patients who had died from sepsis or septic shock were found to have continuous septic foci (101), suggesting a failure to clear the inciting pathogen and/or to eradicate nosocomial infections. This is thought to be a consequence of immunosuppression (99, 100), especially in patients who survive the early hyperinflammatory phase. The reported inability of many septic patients to elicit normal delayed-type hypersensitivity (DTH) skin reactions to standard recall Ags (102) and the frequent reactivation of latent viruses (e.g., cytomegalovirus, Epstein–Barr virus, herpes simplex virus, human herpesvirus-6), sometimes involving multiple viruses at the same time in prolonged sepsis (103), also point to a profound state of immunosuppression.

Multiple other findings lend support to the notion of sepsis-induced immunosuppression. In an earlier study, van Dissel et al. demonstrated that a high ratio of plasma IL-10:TNF-α correlates with increased mortality in febrile patients with community-acquired infection and cautioned against the application of pro-inflammatory cytokine inhibition in sepsis (104). In a separate study, circulatory levels of IL-10 paralleled the sepsis score, and its sustained overproduction was deemed a predictor of severity and fatal outcome (105).

A global cytokine depression has been noted in numerous other studies. After stimulation with lipopolysaccharide (LPS), whole blood samples from septic patients contained less IL-1β, TNF-α, and IL-6 in comparison with samples obtained from non-septic patients admitted for hernia repair or cholecystectomy (106). Munoz et al. reported a profound decrease in the ability of freshly isolated monocytes from ICU patients with sepsis to produce IL-1β, TNF-α, and IL-6 following *ex vivo* exposure to LPS (107). An important finding of this investigation was that monocytes from the survivor subpopulation, but not from those who eventually died from sepsis, regained their cytokine production capacity. Also interestingly, the blunted pro-inflammatory cytokine response was most pronounced in patients with Gram-negative infections. This may be a manifestation of the long-known phenomenon of “endotoxin tolerance,” according to which LPS-exposed cells become refractory to subsequent LPS challenges (108). Endotoxin tolerance arguably serves to protect against uncontrolled inflammation in sepsis, but is also correlated with a high risk of secondary infection and mortality. In septic patients, monocytes are also hyporesponsive to CD40 ligation, which would otherwise result in the upregulation of classic costimulatory molecules B7-1 (CD80) and B7-2 (CD86) and enhanced ability of monocytes to activate T lymphocytes (109). The CD40–CD40L cross-talk does not directly involve the CD14/TLR-4 pathway governing cellular responses to LPS. Therefore, endotoxin tolerance may only partially explain monocyte hyporesponsiveness in sepsis.

Sepsis-induced immunological shortcomings are not limited to leukocytes traveling in the bloodstream. Boomer et al. found that post-mortem splenocytes from septic patients secreted significantly less TNF-α, IFN-γ, IL-6, and IL-10 in response to LPS, CD3/CD28 co-ligation, or stimulation with phorbol 12-myristate 13-acetate (PMA) plus ionomycin when compared with splenocytes from patients who were declared brain dead or those who underwent emergency splenectomy due to trauma (110). Moreover, cytofluorimetric analyses of splenic cell populations revealed signs of T cell exhaustion or anergy. For instance, the frequency of CD4^+^ T cells displaying the anergy/exhaustion marker programmed cell death 1 (PD-1) and that of CD8^+^ T cells expressing the prototype co-inhibitory molecule cytotoxic T-lymphocyte antigen-4 (CTLA-4) were higher in septic than in control patients. Both subsets also expressed low levels of IL-7 receptor α chain (CD127) that promotes cell survival. Consistent with these observations, splenic APCs from septic patients exhibited decreased B7-2 and HLA-DR and increased PD-ligand 1 (PD-L1) levels. It is noteworthy that weak expression of HLA-DR is a common abnormality in sepsis. In fact, measuring monocytic HLA-DR levels has been used to identify an immunosuppressed state in patients with sepsis and septic shock and to monitor the efficacy of sepsis immunotherapy (111).

Boomer et al. also demonstrated that within the post-mortem lung tissues of septic patients, PD-1 expression on CD4^+^ cells and PD-L1 expression on plasmacytoid dendritic cells (pDCs) were augmented in comparison with control lung tissues obtained from transplant donors or cancer resections (110). Finally, this comprehensive study reported two- and three-fold increases in the frequencies of splenic Treg cells and lung myeloid-derived suppressor cells (MDSCs), respectively, in sepsis. Treg cells are relatively resistant to sepsis-induced apoptosis, and their percentage increases also in the circulation of patients with sepsis (99, 112). Using the CLP mouse model, Delano et al. found that GR-1^+^CD11b^+^ MDSCs that produce IL-10 among other cytokines and skew T cell responses toward a Th2 phenotype increase numerically and remain elevated within the spleen, lymph nodes, and bone marrow (113). Therefore, suppressor cell function appears to be a significant component of immunosuppression in sepsis.

Apoptotic death of naïve and adaptive cells of the immune system also contributes to immunosuppression. We detected widespread apoptosis in the spleen of mice with feces-induced peritonitis (FIP), which we used as a model of intra-abdominal sepsis (114). This was due to a profound apoptotic loss of splenic T cells, B cells, NK cells, and macrophages (37). Hotchkiss et al. performed rapid tissue harvesting at the bedside of patients dying from sepsis and demonstrated a marked loss of splenic CD4^+^ T cells, B cells and DCs (115, 116). Felmet and coworkers reported similar depletions, prolonged lymphopenia, and hypocellularity accompanied by apoptosis in the thymus, spleen and lymph node autopsies of pediatric ICU patients with nosocomial sepsis and multiple organ failure (117). Toti et al. found a dramatic depletion of B and T cells in the spleen of preterm and full-term neonates who died of early-onset sepsis due, likely, to *in utero* infection with Gram-positive or -negative microbes (*aka*. chorioamnionitis) (118). These findings indicate that immune effector cell loss during sepsis is a universal phenomenon across all age groups.

Apoptosis causes immunosuppression through multiple mechanisms. First, severe depletion of B and T cells creates “holes in the repertoire” of adaptive lymphocytes. This jeopardizes the ability of the immune system to launch highly specific responses to pathogens. Furthermore, immunological memory cannot be built to protect the survivors at later time points. Apoptosis-mediated shrinkage of the DC compartment not only weakens innate immunity but also contributes to functional T cell inadequacies since naïve T cells can only be primed by DCs. Apoptotic cells are immunosuppressive by nature and their uptake by phagocytic cells can stimulate the release of anti-inflammatory cytokines such as IL-10 (119). In addition, after ingesting apoptotic bodies, DCs may induce death in T cells with which they interact or render them anergic (120). The importance of immune cell apoptosis in the pathogenesis of sepsis can be underscored by the observations that Bcl-2 overexpression or treatment with z-VAD-fmk, a pan-caspase inhibitor, improves survival in mouse models of sepsis (121, 122).

### Immunotherapy for sepsis

Advances in our understanding of the pathogenesis of sepsis have prompted more than 40 clinical trials of immunotherapeutic agents to date. However, the results have been by and large disheartening, with many trials yielding no benefits while a few even aggravated the syndrome, thus leading to their premature termination.

Most previous trials have employed agents that neutralize pathogens or their products [e.g., intravenous immunoglobulin (123) and the anti-endotoxin antibody nebacumab (124)], interfere with pathogen recognition by the host [e.g., the TLR4 antagonist eritoran (125)], or target pro-inflammatory cytokines/mediators [e.g., the anti-TNF-α antibody afelimomab (126) and the recombinant TNF receptor p55–IgG1 Fc fusion protein lenercept (127)] or their receptors [e.g., the IL-1 receptor antagonist anakinra (128) and the platelet-activating factor receptor antagonist lexipafant (129)]. Pro-inflammatory cytokines sometimes exert redundant functions. Therefore, therapeutic approaches targeting individual cytokines are often ineffective. Non-specific corticosteroid therapy has also been used in sepsis, albeit to little avail (130).

Dampening hyperinflammatory responses may benefit some patients in the early phase of sepsis. However, it is now recognized that many others have a global cytokine depression or even a predominance of anti-inflammatory cytokines. Equally important is the fact that most patients rapidly progress to an immunosuppressed state associated with a higher susceptibility to secondary and opportunistic infections, in which case weakening the immune system may be counterintuitive. This may explain, at least partially, the failure of the vast majority of previous trials designed to block inflammatory mediators in sepsis. In fact, apart from prophylactic measures and antibiotic administration, adjuvant therapy to restore immune competence in immunosuppressed septic patients may prove beneficial or even lifesaving (99). In an earlier application of such approaches, Döcke et al. administered IFN-γ to a small cohort of septic patients whose monocytes had reduced HLA-DR expression and whose whole blood cells produced only minute amounts of TNF-α in response to LPS stimulation (131). Treatment with IFN-γ reversed these deficits and also importantly resulted in resolution of sepsis in most cases. In a more recent case report by Nalos and coworkers, successful IFN-γ therapy in a male patient with type-2 diabetes and prolonged, disseminated *S. aureus* sepsis was documented (132).

Granulocyte-macrophage colony-stimulating factor (GM- CSF), a hematopoietic growth factor that stimulates the production of neutrophils and monocytes from bone marrow stem cells, has also been used and shown promise in immunosuppressed septic patients. In a relatively small-scale clinical trial, GM-CSF administration was safe and normalized the expression of monocytic HLA-DR and shortened the duration of mechanical ventilation and hospital/ICU stay due to sepsis (111). In a subsequent study, GM-CSF restored the *ex vivo* TNF-α production capacity of whole blood cells and prevented nosocomial infections in pediatric patients with multiple organ dysfunction syndrome (133).

IL-7 and IL-15 are two other immune-enhancing cytokines with enormous therapeutic potentials. Dubbed as the “maestro of the immune system” (134), IL-7 is a pleiotropic cytokine with diverse biological properties, some of which may correct immunological abnormalities linked to sepsis. Clinical trials of IL-7 in other conditions (e.g., metastatic cancer, HIV-1 infection, and progressive multifocal leukoencephalopathy) have demonstrated that its systemic administration is safe and well tolerated (135–137). Furthermore, it seldom causes fever or significant pro-inflammatory cytokine production. IL-7 induces naïve and memory T cell proliferation without a predilection for Treg cell expansion (138). Therefore, its administration could potentially replenish the T cell pool following drastic lymphocyte depletion in sepsis. IL-7 is known to upregulate the expression of the anti-apoptotic molecule Bcl-2 in T cells, thus promoting their survival (139) and that of cell adhesion molecules (140), thus potentiating leukocyte trafficking into the site(s) of infection. In addition, treatment with IL-7 increases the diversity of the TCR repertoire (138, 139), which in turn improves the breadth of pathogen-specific T cell responses. Together, these activities can immensely help combat pathogens during sepsis. The therapeutic benefit of IL-7 has been validated in CLP. Using this animal model, Unsinger et al. found that recombinant human IL-7 (rhIL-7) can normalize the DTH reaction, block T cell apoptosis, restore IFN-γ production, and improve host survival (140). Similar results were obtained in a “two-hit” model of fungal sepsis in which mice underwent CLP to induce peritonitis followed by an intravenous injection of *Candida* *albicans* (141) to mimic delayed secondary infections in ICU patients. Venet et al. reported that IL-7 plasma levels and CD127 expression by T lymphocytes remain unaltered in septic shock (142). More importantly, T cells from septic patients and healthy volunteers exhibited comparable signal transducer and activator of transcription 5 (STAT5) phosphorylation and Bcl-2 upregulation when exposed to rh-IL-7. In addition, rh-IL-7 augmented T cell proliferation and IFN-γ production by CD8^+^ T cells in response to anti-CD2/CD3/CD28-coated beads that were used *ex vivo* as artificial APCs. Therefore, the IL-7:IL-7 receptor machinery appears to be fully operative in septic patients and may thus be utilized to reverse their immunological impairments.

IL-15 is another pleotropic cytokine involved in the development, maintenance, and proliferative responses of multiple lymphocyte lineages. It optimizes effector and memory CD8^+^ T cell functions under normal conditions and also reportedly controls the homeostatic recovery of naïve CD8^+^ T cells after CLP-induced sepsis (143). Unlike IL-7, IL-15 is a potent promoter of NK cell and DC functions, which can be defective in sepsis. In fact, IL-15 therapy was demonstrated to block NK cell, DC, and CD8^+^ T cell apoptosis, to increase IFN-γ levels in the circulation, and to improve survival of mice rendered septic by the CLP procedure or *Pseudomonas aeruginosa* pneumonia (144). In a recent study, septic patients with severe lymphopenia had low expression of Bcl-2 mRNA in their peripheral blood mononuclear cells despite moderately increased plasma IL-15 concentrations (145). Whether such IL-15 quantities are still insufficient and whether treatment with exogenous IL-15 may help correct immunological incompetence in sepsis warrant further investigation.

Several other studies have focused on blockade of co-inhibitory receptors (e.g., PD-1) to alleviate sepsis-induced immunosuppression. Since the induced expression of PD-1 on T cells was first linked to their exhaustion in the context of chronic viral infection (146), interfering with PD-1:PD-L1 interactions has been viewed as a tempting therapeutic approach to rejuvenating T cells in various conditions including sepsis. Administration of an antagonistic monoclonal antibody (mAb) to PD-1 after the CLP procedure rescued the DTH response and prevented the expression loss of the pro-survival protein Bcl-xL in splenic T cells (147). This was accompanied by a reduction in depletion of lymphocytes and DCs and mortality. In a separate study, treatment with an anti-PD-L1 Ab either before or after CLP led to improved survival of septic mice (148). In addition, PD-L1 blockade prevented the loss of B and T cells, increased blood levels of IL-6 and TNF-α while decreasing IL-10, and lowered bacterial burden in the circulation and within the peritoneal cavity. Therefore, the PD-1:PD-L1 axis is an attractive target for sepsis immunotherapy.

Tailoring immune intervention strategies to patients’ factors and conditions (e.g., age, cytokine profiles, immune competence, co-morbidities) and to the phase of sepsis (i.e., early versus protracted) will improve the likelihood of success (99). Biomarker-guided, personalized therapies that are carefully timed and sufficiently monitored using laboratory and/or clinical measures should prevent short- and long-term, adverse consequences of sepsis. Agents that block inflammatory cytokines need to be short-acting, used in early sepsis, and reserved for a group of patients with drastically elevated pro-inflammatory cytokine levels. On the contrary, adjuvant immunotherapy will benefit septic patients who are in an immunosuppressed state. Failure of leukocytes to produce TNF-α in response to LPS stimulation *ex vivo*, subnormal expression of monocytic HLA-DR, upregulated expression of PD-1 or PD-L1 on circulating leukocytes, infections caused by opportunistic pathogens (e.g., *Candida* spp.) and reactivation of otherwise latent viruses, such as cytomegalovirus and herpes simplex virus, can help identify such patients.

### Animal models of sepsis

Using preclinical models that reliably replicate human sepsis is essential for the development of novel diagnostic biomarkers, prognostic indicators and therapeutic modalities that can be truly translatable from the benchtop to the bedside. Common animal models of sepsis, which are summarized in Table 1, utilize a variety of septic triggers or insults including LPS injection, systemic administration of microbes, surgical disruption of the intestinal barrier integrity, and direct introduction of feces into the peritoneal cavity.

**Table 1:** **Common Animal Models of Sepsis**.

Species	Model	Immunopathology and reported manifestations	Advantages	Disadvantages
Mouse	Endotoxicosis	Rapid but transient inflammatory cytokine response, hypotension (149); leukopenia (150) hypodynamic cardiovascular changes (151); multi-organ injury, mortality within days	Simple and reproducible	Lack of infectious focus; cytokine response magnitude may not represent human sepsis (149); poor reflection of complex physiological/immunological changes of human sepsis
	Systemic bacterial administration	Rapid but transient inflammatory cytokine response when given i.v., slow and sustained inflammatory cytokine response when given i.p. (180); bacteremia, hypotension, hypodynamic cardiovascular changes with infected fibrin clot (181); multi-organ injury, mortality within hours to days	Simple and reproducible	Variability introduced by the choice of bacterial strain and administration route; large bolus of bacteria may not reproduce changes of human sepsis; may reflect endotoxicosis in the case of Gram-negative bacteria
	Host barrier disruption (CLP/CASP)	Rapid pro/anti-inflammatory cytokine response (187) that is more severe in CASP vs CLP (201); polymicrobial bacteremia, hypotension, hyperdynamic cardiovascular changes (188); T and B cell apoptosis, immunosuppression (189); multi-organ injury, mortality within hours to days	Polymicrobial, severity controlled by size of puncture/stent diameter; CLP reproduces immunosuppressive phase	Requires surgical techniques; high experimental variability; abscess formation may prevent disease development (201)
	Feces-induced peritonitis (FIP)	Rapid pro/anti-inflammatory cytokine response, systemic bacterial dissemination, splenocyte apoptosis (114); hypothermia, impaired metabolism, hypodynamic cardiovascular changes (203); mortality within days	Simple, controlled inoculum; reflects polymicrobial peritonitis	Microbial dose and composition of the fecal inoculum often unknown; cytokine response magnitude more severe vs. CLP (206)
Rat	Endotoxicosis	Rapid pro/anti-inflammatory cytokine response (152); hypermetabolism, hypotension, hypodynamic cardiovascular changes with lethal dose i.v. (153); hyperdynamic cardiovascular changes with non-lethal dose i.p. (165); multi-organ injury, mortality within hours to days	Simple and reproducible	Lack of infectious focus, poor reflection of complex physiological/immunological changes of human sepsis
	Systemic bacterial administration	Rapid pro/anti-inflammatory cytokine response (170); hypotension, bacteremia, hypodynamic cardiovascular changes with high dose (171); hyperdynamic cardiovascular changes with low dose (177) and infected fibrin clot (185); mortality within hours to days	Simple and reproducible, can reproduce hyperdynamic changes in human sepsis	Large bolus of bacteria may not reproduce changes of human sepsis; may reflect endotoxicosis in the case of Gram-negative bacteria
	Host barrier disruption (CLP/CASP)	Rapid pro/anti-inflammatory cytokine response (190), hyperdynamic cardiovascular changes (191); polymicrobial bacteremia, leukopenia, thrombocytopenia (192); multi-organ injury, mortality within hours to days	Polymicrobial, severity controlled by size of puncture/stent diameter	Requires surgical techniques; high experimental variability
Rabbit	Endotoxicosis	Rapid inflammatory cytokine response, hypotension, hypodynamic cardiovascular changes with high dose (154); hyperdynamic cardiovascular changes with low dose (164); hypothermia, leukopenia (155); multi-organ injury, mortality within hours to days	Simple and reproducible, increased sensitivity to LPS compared to rodents	More expensive than rodent models; lack of infectious focus, poor reflection of complex physiological/immunological changes of human sepsis
	Systemic bacterial administration	Rapid inflammatory cytokine response, hypotension, leukopenia, thrombocytopenia (172); bacteremia, hypothermia, neutrophil apoptosis (182); multi-organ injury, mortality within hours	Simple and reproducible	More expensive than rodent models; less well-characterized; may reflect endotoxicosis in the case of Gram-negative bacteria
Pig	Endotoxicosis	Rapid pro/anti-inflammatory cytokine response, neutropenia, lymphopenia (156); hypotension, DIC, hypodynamic cardiovascular changes (157); hyperdynamic cardiovascular changes with fluid resuscitation (160); mortality within hours	Simple, reproducible, porcine physiology and LPS sensitivity similar to humans	Expensive housing and care costs; lack of infectious focus; poor reflection of complex physiological/immunological changes of human sepsis
	Systemic bacterial administration	Rapid pro/anti-inflammatory cytokine response, bacteremia, DIC (173); hypotension, hypodynamic cardiovascular changes (174); multi-organ injury, mortality within hours to days	Simple, reproducible, porcine physiology similar to humans	Expensive housing and care costs; large bolus of bacteria may not reproduce changes of human sepsis; may reflect endotoxicosis in the case of Gram-negative bacteria
	Feces-induced peritonitis (FIP)	Inflammatory cytokine response, hypotension, hyperdynamic cardiovascular changes with fluid resuscitation (204, 205); leukocytosis, endotoxemia (174); multi-organ injury, mortality	Porcine physiology similar to humans; reflects polymicrobial peritonitis	Expensive housing and care costs; microbial dose and composition of the fecal inoculum often unknown
Non-Human Primate	Endotoxicosis	Rapid but transient pro-inflammatory cytokine response, hypotension, hypodynamic cardiovascular changes (158); thrombocytopenia, leukopenia (159)	Cross-reactivity with human thera-peutics and diagnostic tools, most comparable to human physiology	Most expensive housing and care costs; ethical concerns; more accurately reflects human endotoxicosis rather than sepsis
	Systemic bacterial administration	Rapid pro/anti-inflammatory cytokine response, hypotension, leukopenia, thrombocytopenia, DIC (175); hypodynamic cardiovascular changes that become hyperdynamic with fluid resuscitation (176); multi-organ injury, mortality within hours to days	Cross-reactivity with human therapeutics and diagnostic tools, most comparable to human physiology	Most expensive housing and care costs; ethical concerns; may reflect endotoxicosis in the case of Gram-negative bacteria

*CASP, colon ascendens stent peritonitis; CLP, cecal ligation and puncture; DIC, disseminated intravascular coagulation; FIP, feces-induced peritonitis; i.p., intraperitoneal; i.v., intravenous; LPS, lipopolysaccharide*.

Clinical and paraclinical (e.g., biochemical) features of sepsis serve as guiding principles for the development of *bona fide* animal models and for validation of their relevance to the human syndrome. Such models should take into consideration both the early hyperinflammatory state, which is characterized by massive pro-inflammatory cytokine production and its consequences (e.g., fever), and the concurrent or subsequent anti-inflammatory responses that contribute to anergy, immunosuppression and susceptibility to secondary and opportunistic infections. Hemodynamic changes sometimes requiring fluid resuscitation, organ damage, apoptotic death of immunocytes, and mortality from sepsis also need to be simulated. Animal models should also ideally permit therapeutic intervention at defined stages of sepsis and efficacy testing of such treatments. Accordingly, gross outcome measurements, such as weight loss and death rates, should be complemented with laboratory assessments of immune competence or incompetence (e.g., cytokine production and anergy/exhaustion marker expression).

Findings from models in which young, adult and otherwise healthy animals are utilized may not accurately represent the “real-life” features of sepsis in the rising elderly populations. This is a major limitation of animal models that place a disproportionate emphasis on sepsis-induced hyperinflammation, which no longer accounts for most deaths due to sepsis at least when advanced ICU facilities and robust practice of critical care are in place. Therefore, experimentation with older animals and those with co-morbidities may provide a more realistic picture of sepsis in vulnerable populations.

One of the most routinely utilized agents to induce sepsis in small and large animals is LPS (149–160), a glycolipid found abundantly in the outer membrane of Gram-negative bacteria. Following intravenous (i.v.) or intraperitoneal (i.p.) injection, LPS binds to the glycosylphosphatidylinositol (GPI)-anchored protein CD14 and signals through TLR-4 to provoke a systemic inflammatory response often referred to as “endotoxicosis” (161, 162). This response is characterized by pro-inflammatory cytokine production, multiple organ injury and hypotensive shock, which are hallmarks of early sepsis. LPS administration is simple and does not require advanced surgical techniques. In addition, its dosage can be easily controlled. However, one should keep in mind that exposure to large amounts of LPS may result in an immediate hypodynamic cardiovascular state that does not represent human sepsis (163). Several groups have overcome this problem by developing models that use sublethal doses of LPS (164, 165) or aggressive fluid resuscitation (166). Also importantly, bolus injection of LPS into laboratory animals triggers a severe inflammatory cytokine response that differs in magnitude and sustenance from what is observed in clinical sepsis (163, 167).

Lethal shock and disseminated intravascular coagulation (DIC) can be induced in mice by two consecutive injections of LPS separated by a 24-h interval (168). In this model, which is known as “generalized Shwartzman reaction,” a “super low” dose of LPS is injected followed by a larger systemic dose that elicits rapid pro- and anti-inflammatory responses, coagulopathy and multi-organ damage. It is noteworthy that the initial priming dose in Shwartzman reaction is smaller than that causing endotoxin tolerance (169). It is believed that tolerizing doses of LPS activate the canonical nuclear factor-κB (NF-κB) pathway leading to robust expression of pro-inflammatory mediators as well as a myriad of suppressive elements designed to prevent progressive inflammation (169). In contrast, super low doses of LPS, such as those used in the priming phase of Shwartzman reaction, fail to activate the NF-κB pathway. Instead, they trigger the activation of CCAAT/enhancer binding protein δ (C/EBPδ) in an IL-1 receptor-associated kinase 1 (IRAK1)-dependent manner resulting in mild but persistent expression of inflammatory mediators (169).

All animal models of endotoxicosis lack an infectious focus. In addition, since LPS is only present in Gram-negative bacteria, these models do not represent polymicrobial sepsis caused by mixed Gram-positive and Gram-negative microbes.

Systemic administration of a large number of bacteria, typically *E. coli*, instigates a massive inflammatory cytokine response, cardiovascular collapse and rapid mortality (170–175). Fluid resuscitation or sublethal dosages of bacteria can be used to better mimic the septic response and its hemodynamic manifestations in humans (176–178). These models allow for bacterial strains and numbers to be carefully chosen and for host responses to develop against intact microbial pathogens. However, they are more similar to models of endotoxicosis than full-blown infections when Gram-negative bacteria are used. Many bacterial strains are complement-sensitive and lysed shortly after they enter the circulation, thus releasing their endotoxin content (179). Moreover, systemic bacterial infusion gives rise to serum TNF-α concentrations that are orders of magnitude larger than those found in septic patients or in peritonitis models (179). Lastly, the route of administration can impact the vigor of the septic response. For instance, a robust but transient TNF-α response is elicited following an i.v. challenge of mice with live *E. coli* O111, whereas an i.p. challenge leads to much lower but more sustained blood levels of TNF-α (180).

Surgical implantation of bacteria (e.g., *E. coli*)-laden fibrin clots into the peritoneum has also been used to induce sepsis in several species (181–185). Some of these models more accurately reproduce the hyperdynamic state and slow, sustained release of cytokines associated with human sepsis.

Cecal ligation and puncture (CLP) is considered by many as the “gold standard” of intra-abdominal sepsis models. This relatively simple surgical procedure involves a laparotomy and ligation of the cecum in a non-obstructing manner followed by puncturing the ligated portion to allow fecal content to leak into the otherwise sterile peritoneal cavity (186). Therefore, a source of necrotic tissue combined with an infectious focus that persistently challenges the host with enteric microbes causes polymicrobial sepsis. CLP-inflicted sepsis resembles the clinical syndrome since it can set in motion a systemic pro-inflammatory cytokine response as well as a compensatory anti-inflammatory response and a hyperdynamic cardiovascular state (162, 187–192). Furthermore, CLP is particularly useful for studying the delayed phase of sepsis in which immune responses are impaired. This is possible by the “two-hit” versions of the model, in which mice undergo CLP and are subsequently challenged with a secondary/opportunistic pathogen, such as *Streptococcus pneumoniae* (193), *Pseudomonas* *aeruginosa* (193–195), *Candida albicans* (141) or *Aspergillus fumigatus* (196). Logistically, the CLP procedure is quick to perform by an experienced experimentalist. It can also be readily modified to investigate varying degrees of inflammation and different survival intervals. The length of ligated cecum (197), the size of the needle used for the perforation (198), and the number of punctures made (199) can all determine the severity of sepsis and the speed with which death occurs. It needs to be noted that the CLP outcome may vary considerably among different laboratories and animals depending upon the experimentalist’s surgical expertise and the animals’ sex, age, strain, housing conditions, cecal content, and even cecal fullness when CLP is performed (167). Another disadvantage of the CLP model lies in the host’s natural ability to form an abscess in order to contain infection (200, 201). Therefore, treatments that promote abscess formation may improve survival in CLP, which may introduce bias by adding a confounding variable in the experiment.

Another model of host barrier disruption leading to sepsis is colon ascendens stent peritonitis (CASP), in which a stent is inserted into the ascending colon to allow for leakage of fecal matter into the peritoneal cavity (201, 202). Although similar to CLP in principle, CASP represents persistent peritoneal infection rather than abscess formation and causes a more robust cytokine response and higher bacterial loads within several organs. The severity of and mortality from CASP are influenced by the diameter of the stent and also by its removal at defined time points. This mimics surgical interventions to eliminate infectious foci in humans.

Host barrier disruption models are heavily reliant on surgical techniques and relatively difficult to standardize. An alternative approach is to simply inject animals with a given amount of fecal solution i.p. (114, 174, 203–205). This is called the feces-induced peritonitis (FIP) model of polymicrobial sepsis, for which we recently developed a robust scoring system (114). Early inflammatory cytokine production in FIP is typically much more intense than that caused by CLP (206). The amount of feces to be injected i.p. can be adjusted to alter the severity and outcome of sepsis. An additional advantage of FIP is that fecal solutions with identical microbial loads and composition can be injected into multiple recipient cohorts. This is in contrast with barrier disruption models requiring the leakage of each animal’s intestinal content into the peritoneal cavity, which is an inevitable source of variation. A limitation of the FIP model is that the dosage and species of bacteria introduced into the recipients are usually unknown given that intestinal flora vary according to the animal strain, commercial source and housing conditions. Finally, the state of immunosuppression that follows the hyperinflammatory phase of sepsis has not been fully characterized in FIP.

Despite the abundance of animal models for sepsis, there is currently no one truly clinically relevant model that fully recapitulates all the complex immunological, hemodynamic, and pathophysiological responses seen in human sepsis. The reason for outcome discrepancies between animal models and clinical sepsis is multifactorial, but partially stems from the heterogeneity of patient populations. Nevertheless, we continue to rely on current animal models and strive to come up with improved models in order to better understand the pathogenesis of sepsis and to design and test novel treatments for this fatal syndrome.

### *i*NKT cells and sepsis

Several groups including ours have explored the effector or regulatory capacities of *i*NKT cells and their synthetic glycolipid agonists in sepsis and endotoxic shock.

Rhee et al. from Alfred Ayala’s laboratory first reported that treating 129S1/SvImJ mice with an anti-CD1d mAb (clone 1B1) before the CLP surgery could reduce plasma and splenic IL-6 and IL-10 levels and prevented sepsis-induced mortality in some of the treated mice (207). They also noted a significant increase in the frequency of cell populations co-expressing T and NK cell markers, which could be reversed by anti-CD1d treatment. It needs to be noted that although the 1B1 mAb has been used extensively to block CD1d interactions with NKT cell TCRs, it may also potentially activate CD1d^+^ APCs (208). Therefore, the mechanism of action of this mAb could not be definitively determined. More importantly, both *i*NKT and *v*NKT cells interact with CD1d (50). We now know that there are other CD1d-restricted T cell types such as a subpopulation of γδ T cells (209) that can be affected by anti-CD1d treatment. Nevertheless, the study of Rhee et al. indicated a role for CD1d-restricted T cells in sepsis and set the stage for subsequent important investigations.

**Figure 1:**
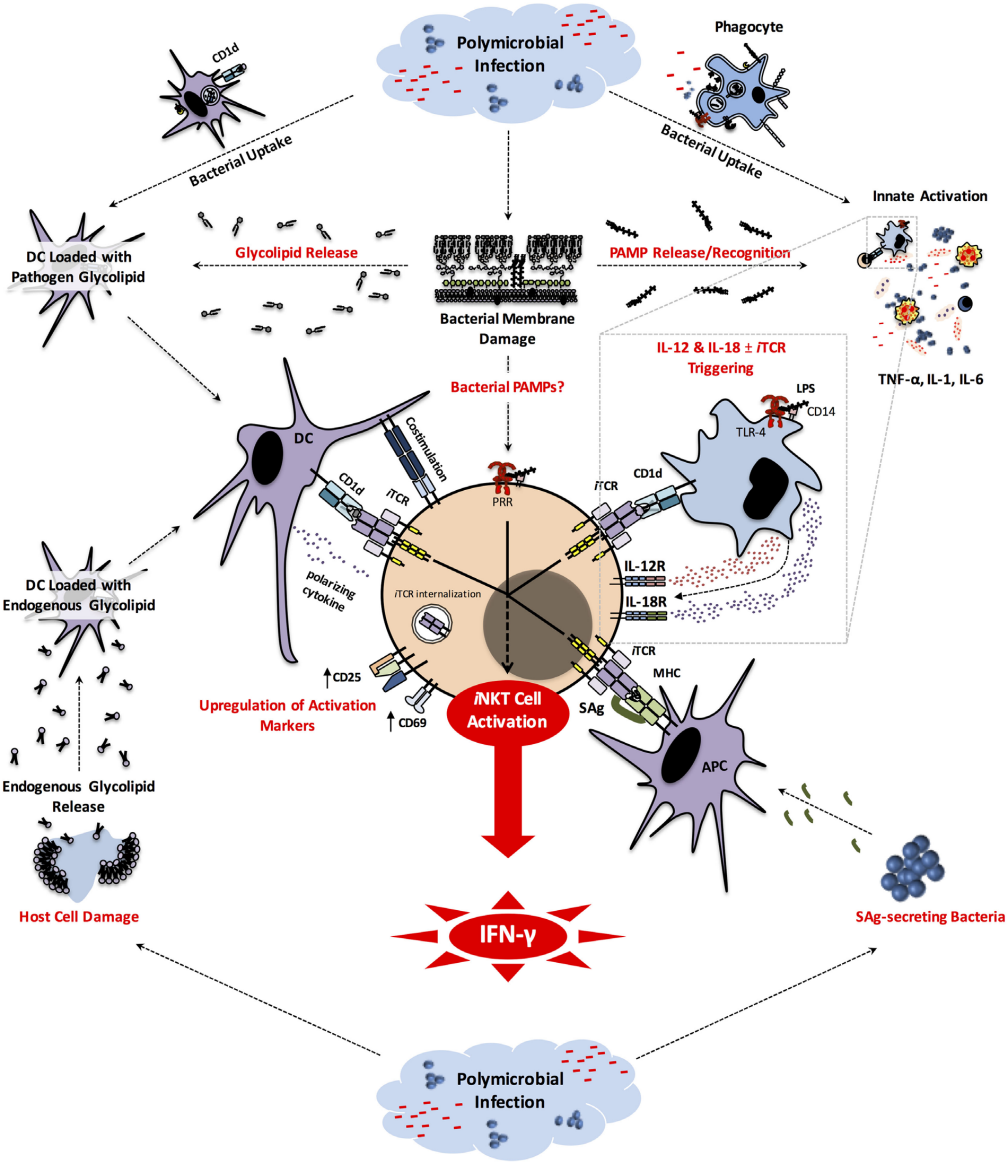
***i*NKT cell activation pathways in sepsis**. Early in the course of the host response to bacterial pathogens involved in sepsis, the engulfment of these microbes by phagocytic cells generates pathogen-derived glycolipids that can be displayed by CD1d to induce *i*NKT cell activation. Phagocytic cells that have taken up bacteria and/or sensed PAMPs (e.g., LPS) through PRRs (e.g., TLR-4) secrete inflammatory cytokines. Some of these cytokines (e.g., TNF-α, IL-1, IL-6) are responsible for clinical manifestations of sepsis, while others (i.e., IL-12 and IL-18) can activate *i*NKT cells. The latter pathway often, but not always, requires CD1d-mediated presentation of endogenous glycolipids to *i*NKT cells. SAg-secreting bacteria, such as *Staphylococcus* spp. and *Streptococcus* spp. participating in Gram-positive bacterial sepsis, can directly activate *i*NKT cells. It is possible that bacterial PAMPs may be detected by *i*NKT cells. Finally, during or as a result of the septic insult, host cell damage leads to release and/or modification of endogenous glycolipids that can be potentially presented by CD1d to trigger *i*NKT cell activation in an *i*TCR-dependent manner. Once activated, *i*NKT cells produce pro-inflammatory cytokines, most notably IFN-γ that plays a pivotal role in sepsis-inflicted immunopathology. APC, antigen-presenting cell; CD, cluster of differentiation; DC, dendritic cell; IFN, interferon; IL, interleukin; *i*NKT, invariant natural killer T cell; *i*TCR, invariant T cell receptor; LPS, lipopolysaccharide; MHC, major histocompatibility complex; PAMP, pathogen-associated molecular pattern; PRR, pattern recognition receptor; TLR, Toll-like receptor.

Hu et al. extended the above study to other mouse strains (210). They demonstrated that pre-treatment of BALB/c mice with 1B1 before CLP confers upon them a survival advantage. This treatment also prevented the rise in circulating levels of TNF-α, IL-6, monocyte chemotactic protein (MCP)-1 and IL-10. Within the liver, mice receiving 1B1 had lower frequencies of NKT cells capable of producing TNF-α, IL-6, IL-4 or IL-10, indicating no bias toward either a pro- or anti-inflammatory phenotype. Interestingly, however, the percentage of IL-6-producing hepatic macrophages declined whereas that of IL-10-producing cells increased upon anti-CD1d treatment. How CD1d contributes to the immunopathology of sepsis is not clear. It is possible that lipid antigens derived from bacterial pathogens are loaded into CD1d and presented to NKT cells. Alternatively or in addition, recognition of pathogen-associated molecular patterns (PAMPs) by PRRs such as TLRs may lead to the production of IL-12 and IL-18 by APCs during sepsis. Once coupled with CD1d-mediated presentation of endogenous lipids, these cytokines can induce NKT cell activation (42, 43). Sepsis-induced tissue injury may also increase, release and/or modify endogenous lipids that can be displayed by CD1d to trigger NKT cell responses (Figure 1). Consistent with this hypothesis, a previous study reported that serial injections of apolipoprotein E (ApoE), a component of plasma lipoproteins, alters NKT cell compartments and increases CLP-induced mortality in rats (211).

Hu et al. also used the CLP model to examine the contribution of the invariant subset of NKT cells to sepsis (210). They found a marked decline in the frequency of hepatic *i*NKT cells, defined by their reactivity with α-GalCer-loaded CD1d tetramer, in both C57BL/6 and BALB/c mice. This was accompanied by upregulation of CD69 and CD25 on the surface of *i*NKT cells indicating their enhanced activation on a per cell basis. There exist several possibilities to explain the lower percentage of detectable *i*NKT cells in the liver of septic mice. These include *i*TCR internalization, which is a well-known phenomenon in the context of *i*NKT cell activation by synthetic glycolipids (212), cell death *in situ*, or migration to other locations. To address these possibilities experimentally, one could assay for intracellular *i*TCRs or quantify mRNA corresponding to the Vα14–Jα18 TCR rearrangement in hepatic non-parenchymal cells, or track *i*NKT cell movements in the body.

To ascertain whether *i*NKT cells play a pathogenic or protective role in sepsis, Hu and coworkers used Jα18^−/−^ mice that lack *i*NKT cells (210). These animals exhibited reduced mortality due to CLP as well as ablated TNF-α, IL-6, MCP-1 and IL-10 systemic responses in comparison with wild-type C57BL/6 mice. It was recently found that the TCRα repertoire of Jα18^−/−^ mice that have been widely available to the research community is shrunk by ~60% (213). Therefore, the cellular deficiency in Jα18^−/−^ mice is not exclusive to *i*NKT cells, which necessitates *i*NKT cell reconstitution experiments to validate results obtained using these animals.

More recently, Heffernan et al. demonstrated that while CLP causes a drop in the frequency of *i*NKT cells in the liver, both the absolute number of *i*NKT cells and their frequency among T lymphocytes are elevated in the circulation and within the peritoneal cavity, which is considered the site of polymicrobial infection in this model (214). Furthermore, a much bigger fraction of peritoneal *i*NKT cells expressed CD69 in septic mice in comparison with the sham laparotomy control group. Although *i*NKT cell mobilization by CLP was not directly monitored, these results support the scenario in which *i*NKT cells migrate out of the liver and toward the source of infection, which should account for their decline in the liver. Intriguingly, this migration was mediated by PD-1, which is well known as an anergy/exhaustion marker. Following sepsis, PD-1^−/−^ mice exhibited a numerical increase in activated hepatic *i*NKT cell populations but intact peripheral blood or peritoneal *i*NKT cell compartments when they were compared with the sham controls. Whether the role of PD-1 in *i*NKT cell migration is intrinsic to these cells was not studied. This question could be addressed by reconstitution of Jα18^−/−^ mice with PD-1-sufficient or -deficient *i*NKT cells prior to the CLP procedure. These investigators also found that once accumulated in the peritoneal cavity, *i*NKT cells influence the ability of local macrophages to phagocytose bacteria and clear infection. Bacterial load in the cavity was lower in septic Jα18^−/−^ mice than in wild-type controls. In addition, peritoneal macrophages derived from septic Jα18^−/−^ mice were more potent than those from septic wild-type animals in engulfing *E. coli*. Collectively, the work of Heffernan and coworkers reveals an interesting interplay between migrant *i*NKT cells and macrophages residing within foci of infection during sepsis. It also suggests that blockade of PD-1 may not only reverse T cell exhaustion to relieve sepsis-induced immunosuppression but also likely benefits the host by modulating the migration capacity of *i*NKT cells to further facilitate microbial clearance.

Taken together, the above studies indicate a pathogenic role for *i*NKT cells in CLP-induced sepsis. Several groups have reached the same conclusion using animal models of LPS-inflicted pathology or lethality. Koide et al. established a link between the resistance of d-galactosamine (d-GalN)-sensitized NC/Nga mice to LPS and the presence of fewer NKT cells in these animals (215). This old protocol utilizes the hepatotoxic agent d-GalN to sensitize laboratory animals to very low doses of LPS, and has been used extensively as a model of endotoxic shock and Gram-negative microbial sepsis. We are of the opinion that the d-GalN sensitization model simulates acute hepatic failure more closely. Nevertheless, participation of inflammatory mediators is evident in its immunopathology amid severe liver damage. While co-administration of d-GalN and LPS led to 100% mortality in C57BL/6 mice within 12 h, it failed to kill NC/Nga mice even at 24 h (215). It also raised the activity level of alanine aminotransferase (ALT) in the serum and that of caspase-3 in the liver extract of C57BL/6 mice but not NC/Nga mice (216). Moreover, drastic lesions with hemorrhage and many apoptotic cells were observed in C57BL/6 but not in NC/Nga liver sections. NC/Nga mice injected with d-GalN and LPS had negligible levels of ­IFN-γ protein in their serum or IFN-γ mRNA in their liver. This was accompanied by a 10-fold reduction in the size of CD3ϵ^+^DX5^+^ NKT cell compartment in the liver although NKT cells were capable of producing ample IFN-γ on a per cell basis. Finally, administration of recombinant IFN-γ to d-GalN-sensitized NC/Nga mice rendered them susceptible to LPS-induced ­mortality. In this body of work, frequency analyses were ­performed on NKT cells co-expressing T and NK cell markers. However, *i*NKT cells are the likely culprits and the early ­triggers of pathology in d-GalN/LPS-prone mice. This is because *i*NKT cells comprise the vast majority of hepatic NKT cells in mice (217). Second, when Koide and coworkers injected NC/Nga mice with the *i*NKT cell superagonist α-GalCer a few hours before the d-GalN/LPS challenge, endogenous IFN-γ production was restored leading to increased expression of inducible nitric oxide synthase (iNOS), appearance of apoptotic cells in the liver, and 100% mortality (215). It was therefore concluded that the ­resistance of NC/Nga mice to the LPS-mediated lethality with d-GalN sensitization is due to impaired IFN-γ production caused by a shortage of *i*NKT cells and reduced nitric oxide ­production in these animals. An additional confirmatory approach would have been to adoptively transfer a large number of syngeneic *i*NKT cells into NC/Nga mice to increase their frequency before testing the susceptibility of the recipients to d-GalN/LPS.

In a different model of endotoxic shock, α-GalCer injection sensitized wild-type mice, but not Jα18^−/−^ mice, to LPS-mediated lethality (218). Interestingly, shock in these animals was accompanied by severe lesions and hemorrhage, marked accumulation of polymorphonuclear leukocytes and mononuclear cells, and significant cell death almost exclusively in the lungs. Although serum ALT levels were elevated, hepatic lesions were focal and mild, and other organs showed no signs of overt injury or other changes except for congestion. Pulmonary manifestations and lethal shock in this model could not be induced by simultaneous administration of α-GalCer and LPS, and required an interval period of 3–24 h between α-GalCer sensitization and the LPS challenge. This is consistent with the kinetics of IFN-γ secretion in response to α-GalCer, which is potentiated by *i*NKT cells and largely contributed by transactivated NK cells (219). Ito et al. found that α-GalCer injection gives rise to high blood levels of IFN-γ within the above timeframe and augments the subsequent production of TNF-α, a major mediator of endotoxic shock, in response to LPS (218). They further demonstrated that neutralizing IFN-γ or genetic deficiency of TNF-α abolishes the systemic lethal shock in this model. Therefore, it was proposed that IFN-γ and TNF-α play pivotal roles in preparation and execution of LPS-mediated lethality, respectively, in α-GalCer-primed mice. Following up on these findings, Tumurkhuu et al. found that priming with α-GalCer increases the frequency of NKT cells among pulmonary non-parenchymal leukocytes and induces local IFN-γ production (220). This resulted in expression of several adhesion molecules, most notably vascular cell adhesion molecule-1 (VCAM-1), on vascular endothelial cells of the lungs, which in turn promoted the accumulation of very late activating antigen-4 (VLA-4)^+^ cells among inflammatory cell recruits in the lungs. This was significant because an anti-VCAM-1 mAb partially averted LPS-mediated lethal shock in α-GalCer-sensitized mice.

In the above studies, the relative contributions of *i*NKT and NK cells to IFN-γ production was not determined. There currently exists no commercially available antibody for selective depletion of *i*NKT cells although online literature search through the World Wide Web indicates that a mAb called NKT14 may serve this purpose in the future. Until this or similar antibodies become available, one could employ anti-asialo GM1 antiserum and an anti-NK1.1 mAb (clone PK136) in parallel cohorts of mice to address this question. The former depletes NK cells without affecting the NKT cell population, and the latter depletes both NK and NKT cells (45, 221).

Another important question is why LPS-induced pathology in α-GalCer-sensitized mice is restricted to the lungs while the liver is largely spared. This is particularly interesting in light of the observation that α-GalCer induces IFN-γ production by both hepatic and pulmonary *i*NKT cells and that IFN-γ is readily detectable at mRNA and protein levels in both organs. It has been argued that IFN-γ signaling is fully operational in the lungs but not in the liver of α-GalCer-primed mice (222). Augmented expression of phosphorylated STAT1 was more sustained in the lungs than in the liver. In addition, IFN regulatory factor 1 (IRF1) was upregulated in the lungs but not in the liver of α-GalCer-treated mice. Second, pulmonary NKT cells reportedly produce IFN-γ as their main cytokine, whereas hepatic NKT cells produce IFN-γ, IL-4 and IL-10. Neutralization of IL-4 enhances STAT1 activation, exacerbates the hepatic injury, and increases the number of apoptotic cells in the liver. Therefore, IL-4 has been proposed to inhibit IFN-γ signaling in the liver while its absence promotes IFN-γ-mediated pathology in the lungs (222). Finally, one might wonder why a potentially similar mechanism is not at play to protect NC/Nga livers in the d-GalN/LPS model (215). It is possible that the cytokine profile of α-GalCer-primed NC/Nga mice differs from that of C57BL/6 and BALB/c mice. The hepatotoxic nature of the d-GalN insult may also mask the influence of other factors involved. These possibilities are not mutually exclusive.

Several studies have focused on the role of *i*NKT cells in systemic Shwartzman reaction. IFN-γ is considered a key cytokine in the pathogenesis of Shwartzman reaction because it induces massive production of TNF-α, IL-1 and other inflammatory mediators. Dieli et al. found Jα18^−/−^ mice on either C57BL/6 or BALB/c background to be resistant to the LPS-induced mortality of Shwartzman reaction (223). Jα18^−/−^ mice had lower serum levels of IFN-γ and TNF-α in comparison with wild-type animals, and administration of recombinant IFN-γ was sufficient to prime these animals. In two more recent studies, Sierci et al. tested the effect of α-GalCer treatment at different time points before or after LPS priming (224, 225). When α-GalCer was given approximately 6, 9, or 12 days prior to the first injection of LPS, mice survived the subsequent LPS challenge and their protection was associated with reduced serum levels of IFN-γ and TNF-α and hepatic level of MCP-1 (224). In stark contrast, when administered 1 or 3 days before priming, α-GalCer failed to protect the mice from lethal endotoxic shock. It appears likely that earlier α-GalCer injection induces *i*NKT cell anergy (226), thus hampering their IFN-γ production capacity. It would be interesting to examine the expression level of PD-1 on *i*NKT cells obtained from α-GalCer-pretreated mice or to test whether blockade of the PD-1:PD-L1 interaction restores Shwartzman reaction. In a separate study, Sierci et al. found that α-GalCer administered within 2 h before or after the LPS challenge rescues the mice (225). This timeframe is consistent with the period in which IL-4 production by α-GalCer-stimulated *i*NKT cells reaches its peak while only minute amounts of IFN-γ are detectable in the serum. Accordingly, Sierci and coworkers noted increased IL-4 and IL-10 responses and decreased levels of IFN-γ and TNF-α in protected mice. In addition, blood levels of ALT and aspartate aminotransferase (AST) were lower in these animals indicating milder injury to the liver. The beneficial effect(s) of Th2-type cytokines were confirmed when mice receiving either an anti-IL-4 or an anti-IL-10 mAb succumbed to endotoxic shock. Therefore, inducing Th2-skewed *i*NKT cell responses may have potential therapeutic applications in sepsis. We recently put this hypothesis to the test by using Th2-promoting *i*NKT cell agonists in the FIP model of sepsis (read below).

In a prospective study, we demonstrated that patients with sepsis have a significantly elevated ratio of peripheral blood *i*NKT:T cells in comparison with non-septic trauma patients (37). The patient cohorts were similar in age and in severity of illness that was calculated based on their Acute Physiology and Chronic Health Evaluation II (APACHE II) scores in the initial 24-h period post-diagnosis (227). Next, we compared wild-type and Jα18^−/−^ mice receiving a fecal slurry i.p. for severity of FIP using a murine sepsis score (MSS) that we recently developed (114) and also for mortality from sepsis. The severity of sepsis was significantly lower in Jα18^−/−^ mice than in wild-type controls. In addition, intra-abdominal fecal challenge resulted in 100% mortality in wild-type animals but no death in septic Jα18^−/−^ mice within 24 h. Importantly, reconstitution of Jα18^−/−^ mice with *i*NKT cells before the septic insult increased the severity of their symptoms. Together, these results confirm the pathogenic nature of *i*NKT cells in the FIP model. In the next series of experiments, we explored the therapeutic potentials of OCH, a Th2-polarizing analog of α-GalCer (34), in FIP. We found that a single i.p. injection of OCH within 20 min after the fecal challenge reduced the MSS scores and prolonged the survival of septic mice compared with vehicle- or α-GalCer-treated animals. These changes were associated with elevated blood levels of the Th2-type cytokine IL-13 and reduced levels of the pro-inflammatory cytokine IL-17. Furthermore, OCH treatment decreased the number of apoptotic T cells, B cells and macrophages in the spleen. Anti-inflammatory mechanisms are known to contribute to sepsis-induced immunosuppression, which may make a septic individual susceptible to opportunistic infections (93). Therefore, we asked whether OCH treatment worsens the microbial load in septic mice. Much to our satisfaction, this was not the case, and blood and tissue homogenates prepared from the heart, lungs, kidneys, liver and spleen of vehicle-, OCH- and α-GalCer-treated septic mice had comparable numbers of microbial colony-forming units. Finally, administration of C20:2, another glycolipid that is even more potent than OCH in inducing a Th2 bias (228, 229) and that additionally suppresses downstream NK cell function (230), also mitigated the severity of FIP-induced sepsis. However, this effect was only transient, which may be explained by the relatively short half-life of C20:2 compared with OCH (228, 230). Based on these results, we propose that Th2-skewing agonists of *i*NKT cells may be employed to treat the hyperinflammatory phase of sepsis without compromising the patient’s immunity to microbial pathogens.

Finally, during polymicrobial sepsis, common bacterial pathogens, such as *Staphylococcus* spp. and *Streptococcus* spp., are likely to release the SAgs they harbor (Figure 1). We recently discovered that staphylococcal and streptococcal exotoxins belonging to phylogenetic group II SAgs can directly activate mouse and human *i*NKT cells leading to IFN-γ production (45). However, anticipating the net effect is not simple because: (i) the type of microbial pathogens involved in sepsis may vary substantially among different individuals; (ii) how multiple SAgs released by multiple bacterial pathogens may cross-regulate the response to each other is far from clear; (iii) host responses to SAgs may be modulated by cell wall components of the very bacteria that release SAgs as we previously described (231).

To summarize, the studies highlighted in this section all point to a pathogenic role for *i*NKT cells in sepsis regardless of the experimental model employed, and IFN-γ is a major mediator of *i*NKT cell-inflicted damage in this context.

### *v*NKT cells and sepsis

The extent to which *v*NKT cells contribute to or regulate sepsis is unknown. However, *in vivo* treatment with sulfatide, a CD1d-restricted ligand of *v*NKT cells, has been demonstrated to attenuate the magnitude of the septic response, thus providing indirect evidence for a protective role of activated *v*NKT cells in sepsis.

The beneficial effect of sulfatide was initially noted in two relatively old studies on LPS-induced sepsis with a focus on how this glycolipid influences leukocyte adhesive properties and transendothelial migration as opposed to NKT cell functions (232, 233). Higashi et al. reported that while 75% of C3H/HeN mice died within 2 days of injection with a large dose of LPS, only 20% of mice that were pretreated with bovine brain-extracted sulfatide succumbed even after 7 days (232). Administration of sulfatide to either C3H/HeN or C57BL/6 mice also partially inhibited their TNF-α response to a sublethal dose of LPS. Finally, using a mouse model of endotoxin-induced hypotension, these investigators demonstrated that treatment with sulfatide prior to the LPS challenge prevents an otherwise progressive decline in systolic blood pressure. Squadrito and coworkers explored the effect of sulfatide on acute lung injury in a rat model of endotoxic shock (233). When administered shortly after the LPS injection, sulfatide was able to partially offset hypotension, revert leukopenia, and diminish myeloperoxidase activity in the lungs that was used as an indication of neutrophil accumulation in this tissue. Also importantly, sulfatide treatment caused a near complete prevention of LPS-induced lethality.

The only published report to date addressing a link between CD1d-restricted, sulfatide-reactive T cells and sepsis has utilized an experimental mouse model of *S. aureus* infection (234). In this work, Kwiecinski et al. found that TCRβ^+^NK1.1^+^α-GalCer/CD1d tetramer^−^ cells, which should contain a sizeable population of *v*NKT cells, have an activated phenotype as judged by their upregulated expression of CD69 in infected mice. They also showed that treating wild-type C57BL/6 mice with porcine sulfatide before bacterial inoculation lowers their blood levels of TNF-α and IL-6 without altering the staphylococcal burden in blood, liver or kidneys. Therefore, sulfatide treatment does not impede the ability of the immune system to combat this pathogen. In fact, wild-type mice receiving sulfatide 1 h before and 3 days after bacterial inoculation were partially protected. Importantly, the survival advantage conferred by sulfatide treatment could be recapitulated in Jα18^−/−^ (*v*NKT-sufficient, *i*NKT-deficient) mice but was missing in CD1d^−/−^ (*v*NKT- and *i*NKT-deficient) mice. The significance of this finding is two-fold. First, it strongly suggests that activated *v*NKT cells mediate the protective effect of sulfatide in septic wild-type mice. Second, unlike in other models where sulfatide treatment induces *i*NKT cell anergy to ameliorate inflammation and injury (235, 236), its beneficial effect in *S. aureus* sepsis does not require the presence of *i*NKT cells. Of note, late injection of sulfatide in this model (i.e., on day 3 post-bacterial inoculation) failed to improve survival. This may be viewed as an impediment to the possibility of sulfatide therapy in staphylococcal sepsis once the symptoms have developed. However, more comprehensive studies are warranted to possibly find a window of opportunity during which sulfatide-based interventions may be effective in staphylococcal and other forms of sepsis.

### MAIT cells and sepsis

MAIT cells are relatively frequent among human innate-like T cells and capable of responding to a wide variety of bacterial and fungal pathogens. The ability of MAIT cells to rapidly produce inflammatory cytokines, together with their strategic positioning at the host–pathogen interface, makes them an ideal candidate to fulfill the role of emergency responders to infection and/or regulators of the septic response.

Grimaldi et al. recently explored how sepsis may change the frequency and absolute number of MAIT cells in the circulation (237). In a prospective study, they recruited a relatively large number of patients with severe sepsis or septic shock and compared their peripheral blood MAIT, *i*NKT, and γδ T cell compartments with those of critically ill patients with non-septic (mostly cardiogenic) shock, and age-matched healthy volunteers. Septic patients exhibited an early and dramatic decrease in their MAIT cell count compared with non-infected critically ill patients or healthy controls. This was unlike *i*NKT or γδ T cell counts that remained unaltered in different groups. Also interestingly, there was no association between MAIT cell and total lymphocyte counts, suggesting that MAIT cells follow an independent kinetic pattern in sepsis. By the same token, the frequency of MAIT cells among CD3^+^TCRγδ^−^ conventional T cells was significantly lower in septic patients than in healthy subjects.

The above investigation also led to other potentially important observations. First, the early drop in MAIT cell frequencies was more pronounced in septic patients with non-streptococcal infections than in those with streptococcal infections. In addition, in a small cohort of patients with severe viral infections in the absence of concomitant bacterial infections, MAIT cell values were similar to those of healthy controls. *Streptococcus* spp. and viruses are known not to activate MAIT cells (238). Therefore, the above findings are consistent with the hypothesis that the observed numerical change in the MAIT cell compartment of septic patients is dictated by the type of pathogen(s) encountered. Second, Grimaldi et al. found a higher cumulative incidence of ICU-acquired infections in patients with a persistent decline in peripheral blood MAIT cells. In fact, patients who did not develop secondary infections showed a gradual return to normal MAIT cell values. Therefore, sepsis-induced changes in the MAIT cell compartment seem to be reversible by nature.

It is not clear why MAIT cell numbers drop early in sepsis. Apoptotic cell death, TCR internalization, and migration to peripheral tissues, for instance toward the infectious focus/foci, may provide an explanation for this phenomenon. The latter possibility is supported by the observation that in a few patients registered in the above study, a higher proportion of MAIT cells was detectable in unspecified biological fluids than in blood (238). This is reminiscent of previous reports that MAIT cell numbers drop in the peripheral blood but increase in the lungs of patients with tuberculosis (238, 239). Monitoring MAIT cell frequencies in individual septic patients and future mechanistic studies to uncover the cause of MAIT cell decline in the blood circulation will be informative.

## Outstanding Questions and Concluding Remarks

Despite decades of active research and numerous clinical trials, sepsis continues to take its Toll on human lives and cause significant morbidity, thus imposing a heavy burden on human populations and healthcare systems worldwide. Standardized management protocols and better ICU facilities have improved sepsis outcomes. However, there is currently no “cure” for this devastating syndrome. Targeting conventional T cells, APCs or individual inflammatory cytokines has not met with success. We propose that NKT and MAIT cells provide attractive targets for immunotherapy of sepsis. This is because: (i) they are abundant in certain anatomic locations where microbial pathogens are first encountered. For instance, *i*NKT cells are enriched in the human omentum (16), for which the term “policeman of the abdomen” was coined by a British surgeon, Rutherford Morison, in 1906 (240). Human MAIT cells may serve as “gate-keepers” in mucosal layers and within the liver where they are highly abundant (66); (ii) NKT and MAIT cells can be activated early in the course of sepsis; (iii) they produce large quantities of immunomodulatory cytokines that control the function of downstream innate and adaptive effector cells, thus setting the tone for subsequent host responses; (iv) NKT and MAIT cells are restricted by monomorphic Ag-presenting molecules (i.e., CD1d and MR1, respectively) as opposed to distinct HLA allomorphs. Therefore, they can be stimulated and potentially manipulated by universal ligands in many, if not most, septic patients. This saves time and allows for therapeutic interventions to be implemented speedily; (v) using Th1- or Th2-polarizing ligands, typically in the case of *i*NKT cells, provides flexibility in tailoring therapies to sepsis stages in which hyper- or hypoinflammatory responses predominate.

Evolutionary conservation of CD1d and MR1 recognition across mammalian species makes animal models of sepsis particularly useful for studying NKT and MAIT cells. There is an urgent need for more investigations in models that mirror aging and various co-morbidities. Humanized mouse models should also shed mechanistic light on how clinical sepsis is initiated, perpetuated, or regulated. Such models could also potentially address some of the discrepancies noted between the results of rodent and human studies on NKT/MAIT cell frequencies, effector functions and homing properties.

Currently available Jα18^−/−^ mice that have been used extensively by many investigators including us were recently found to lack T cells other than *i*NKT cells (213). Therefore, if based merely on Jα18^−/−^ mice, the findings of preclinical studies on sepsis need to be revisited. Antibody-mediated depletion of NKT and NK cells in parallel (45, 221) and functional inactivation of *i*NKT cells by carefully timed α-GalCer treatment (226) are among other experimental options to study these cells *in vivo*. Using Jα18^−/−^ and CD1d^−/−^ mice reconstituted with *i*NKT cells should help solidify our knowledge of the roles that these cells play in sepsis. Reconstitution with CD4^+^ or double-negative subsets or with *i*NKT cells isolated from different tissues will enable functional studies on these cells in the context of sepsis. This is particularly important in light of reported functional differences between various *i*NKT cell subpopulations in other conditions, such as cancer (17). Even if/when mouse models of *i*NKT cell deficiency allow for relatively convincing conclusions to be drawn, one has to remain cognizant of the possibility that *i*NKT cells may behave differently in their steady and activated states (241). Much remains to be learned about direct activation of *i*NKT cells by microbial glycolipids and SAgs likely secreted during polymicrobial sepsis. Future studies should also explore the therapeutic potentials of *i*NKT cell glycolipid agonists when used in combination with antibiotics.

There is a paucity of information on the role of *v*NKT cells in sepsis. This is due, at least largely, to a lack of powerful or stable experimental tools to study these cells. CD1d^−/−^ mice are devoid of both *v*NKT and *i*NKT cells (50). Once exclusively *i*NKT cell-deficient mice become available, they can be used in parallel with CD1d^−/−^ animals to address the relative contribution of *i*NKT and *v*NKT cells to the septic response. Sulfatide-loaded CD1d tetramer reagents invented by Vipin Kumar’s laboratory provide a very useful tool for detection of *v*NKT cells (46) but are not very stable. Treatment with native sulfatide has been used as a means of *v*NKT cell activation *in vivo*. However, it is likely that sulfatide exerts other effects and engages other cell types in the body. Sulfatide-reactive, CD1d-restricted γδ T cells have been described in human (242, 243), but their presence in mice is not completely clear. Future studies will need to test the effect of other *v*NKT cell ligands on sepsis. One such ligand is lysophosphatidylcholine whose levels are in fact altered during inflammatory processes (244). Finally, *v*NKT and *i*NKT cells are known to cross-regulate each other in tumor models (245). Whether a similar cross-talk exists during sepsis remains an open question.

Exploration of MAIT cell roles in sepsis is still in its infancy. Mouse and human MAIT cells have distinct tissue distribution and frequencies. However, inducing sepsis in MAIT cell-deficient MR1^−/−^ mice may still provide useful clues toward understanding the role of these cells in sepsis. In addition, mouse and human MR1 tetramer reagents (68), once more widely available, will undoubtedly advance the field of MAIT cell immunology. They will enable mechanistic and functional studies on MAIT cells and elucidate their effector and/or regulatory functions during sepsis.

CD1d- and MR1-restricted T cells have become a focus of intense investigation in recent years. The advent of novel and reliable tools, techniques and models by which to study these cells will better our understanding of their basic biology and therapeutic potentials in various disorders including sepsis. We remain optimistic that the remarkable, quick-acting and wide-ranging immunomodulatory functions of these cells can be harnessed to invent efficacious treatments for different stages of sepsis.

## Conflict of Interest Statement

The authors declare that the research was conducted in the absence of any commercial or financial relationships that could be construed as a potential conflict of interest.
